# Evidence for Direct Geographic Influences on Linguistic Sounds: The Case of Ejectives

**DOI:** 10.1371/journal.pone.0065275

**Published:** 2013-06-12

**Authors:** Caleb Everett

**Affiliations:** Department of Anthropology, University of Miami, Coral Gables, Florida, United States of America; Stony Brook University, United States of America

## Abstract

We present evidence that the geographic context in which a language is spoken may directly impact its phonological form. We examined the geographic coordinates and elevations of 567 language locations represented in a worldwide phonetic database. Languages with phonemic ejective consonants were found to occur closer to inhabitable regions of high elevation, when contrasted to languages without this class of sounds. In addition, the mean and median elevations of the locations of languages with ejectives were found to be comparatively high. The patterns uncovered surface on all major world landmasses, and are not the result of the influence of particular language families. They reflect a significant and positive worldwide correlation between elevation and the likelihood that a language employs ejective phonemes. In addition to documenting this correlation in detail, we offer two plausible motivations for its existence. We suggest that ejective sounds might be facilitated at higher elevations due to the associated decrease in ambient air pressure, which reduces the physiological effort required for the compression of air in the pharyngeal cavity–a unique articulatory component of ejective sounds. In addition, we hypothesize that ejective sounds may help to mitigate rates of water vapor loss through exhaled air. These explications demonstrate how a reduction of ambient air density could promote the usage of ejective phonemes in a given language. Our results reveal the direct influence of a geographic factor on the basic sound inventories of human languages.

## Introduction

It is generally assumed that the worldwide variation of sounds in human languages is largely arbitrary [1], [2]. That is, cross-linguistic disparities in phonological patterns are assumed to be primarily due to stochastic variation in the phonetic gestures relied upon in particular languages. Diachronic influences resulting from linguistic affiliations, both areal and familial, do yield some tendencies in the regional distributions of phonological patterns. In addition, some linguistic sounds are more common due to their relative ease of articulation or perceptual salience. Nevertheless, cross-linguistic phonetic and phonological variation is presumed to be fundamentally arbitrary in the sense that it is not due to nonlinguistic influences such as the geographic context in which a language is produced. One recent strand of research, however, has challenged this basic assumption by offering compelling evidence that warmer climates correlate positively with the degree of sonority of a given language, at least in a small though diverse sample of about sixty languages [2], [3], [4]. According to such work, more sonorous phonological features (such as simple syllables with higher rates of vowel occurrence and greater mean amplitude) are more likely to occur in languages spoken in warmer climates, putatively because cultures in hotter places rely more heavily on communication at greater distances. Assuming this pattern of sonority holds for larger samples of the world’s languages, its geographic impetus is indirect since the true motivation is supposedly relative proximity of interlocutors during typical communicative events. The pattern is also claimed to relate to factors such as terrain type and flora density, as well as cultural variables such as degree of sexual expressiveness [2]. The *direct* influence of a geographic variable on a language’s sound system has yet to be demonstrated. Here we offer evidence for a direct geographic effect on arguably the most basic facet of phonology, the inventory of phonemes in a given language. This evidence is based on the analysis of data from 567 languages, or approximately 8% of the world’s estimated total of 6,909 languages [5].

We hypothesized that, if geographic factors do somehow directly impact phonemic inventories contra the common assumption in linguistics, the factor most likely to have such an impact would relate to atmospheric conditions. In particular, we speculated that atmospheric pressure might impact the production of non-pulmonic sounds, which do not rely on air egressed from below the larynx. More specifically, we generated the following heuristic conjecture: Ejective phonemes might be more likely to occur in areas of high elevation. This guiding hypothesis was based on simple physical modeling of the vocal tract, discussed below. In short, we speculated that the articulation of ejective consonants might be facilitated by reduced atmospheric pressure. These sounds are the only egressive non-pulmonic sounds in human languages, and involve the compression of air in the pharyngeal cavity, typically via the elevation of a closed glottis [6]. Since atmospheric pressure is reduced at higher elevation, we speculated that this compression would be more easily achieved in locations of relatively high elevation. The evidence we present is consistent with our initial hypothesis, though as we note below there are at least two plausible explanations for the geographic-phonetic correlation we have uncovered.

### Analysis

In order to test the hypothesis that the presence of ejective consonants correlates positively and significantly with elevation, we analyzed the locations and elevations of all languages for which data are provided in Maddieson’s typological database of glottalized consonants including ejectives [7]. The database represents the most comprehensive survey of such sounds and was designed so as to fairly represent all world regions while avoiding overreliance on any particular language families. The geographic component of our data collection and analysis was carried out via Google Earth and ArcGIS v. 10.0, after importing the languages’ coordinates from the database into these programs.

As is noted in one prominent survey of world regions of high elevation, only approximately 15% of the world’s occupied surface area is located at high altitude, typically defined as elevation exceeding 1500 m above sea level [8]. Less than 10% of the world’s population resides in such high altitude areas, and the median person resides at 194 m [8]. Despite the fact that only 15.6% of inhabited land lies within 100 m elevation of the sea, some 33.5% of people live on lands below 100 m. This tendency has become even more pronounced since the publication of [8], as a majority of the world’s largest and growing metropolitan areas are found at or near sea level. Clearly humans tend to gravitate towards lower-lying areas, with relatively few people living in areas of high elevation.

The most significant areas of high elevation on the earth’s inhabitable surface are located in major mountain ranges and associated plateaus. While many mountain peaks over 1500 m in height exist, the large inhabitable areas surrounding the associated mountains are often not themselves at high elevation. This is true, for instance, in the case of some peaks in the Alps and New Guinea. In fact, the vast majority of the world’s inhabitable high altitude surface area is found in six non-contiguous regions that include the world’s largest high elevation plateaus. These regions consist of (1) the North American cordillera, including the Rocky Mountains, Colorado plateau, and the Mexican altiplano, (2) the Andes and the Andean altiplano, (3) the southern African plateau, (4) the plateau of the east African rift and the Ethiopian highlands, (5) the Caucasus range and the associated Javakheti plateau, and (6) the massive Tibetan plateau and adjacent plateaus, most notably the Iranian plateau. In the case of the southern African plateau, one large region in the east and a smaller one in the west exceed 1500 m. Two large regions of the East African rift generally exceed 1500 m (though one of these is divided by a section below 1500 m), yielding a total of four main areas above 1500 m in the case of the African continent. While these are not the only regions of the earth with elevations greater than 1500 m, they represent the bulk of the high elevation surface area inhabited by humans and are readily apparent in charts of polygons exceeding 1500 m, for instance the one provided in [8].

## Results

**Figure 1:**
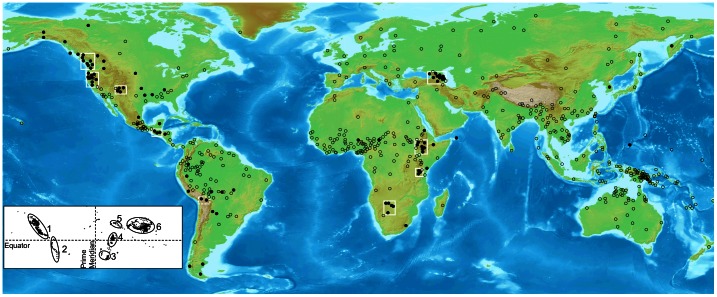
Plot of the locations of the languages in the sample. Dark circles represent languages with ejectives, clear circles represent those without ejectives. Clusters of languages with ejectives are highlighted with white rectangles. For illustrative purposes only. Inset: Lat-long plot of polygons exceeding 1500 m in elevation. Adapted from Figure 4 in [8]. The six major inhabitable areas of high elevation are highlighted via ellipses: (1) North American cordillera (2) Andes (3) Southern African plateau (4) East African rift (5) Caucasus and Javakheti plateau (6) Tibetan plateau and adjacent regions.

The locations of the languages in our sample are plotted in Figure 1. (The world’s major regions of high elevation are plotted in the inset of the figure.) For the sake of clarity, a large portion of the Pacific Ocean is omitted from the figure and, as a result, a handful of the 567 language locations are not depicted. The language locations are based on the latitude and longitude coordinates offered in [7], which were chosen in accordance with the location-finding criteria relied upon by the World Atlas of Linguistic Structures (WALS), of which Maddieson’s rich survey represents one chapter [9]. Since languages are treated as individual data points through these criteria, the geographic distributions of some widespread languages are treated as singular locales that reflect in many cases the larger area in which they developed. For instance, English is represented via one location in England only. In the vast majority of cases, languages are in fact spoken in relatively constricted areas geographically. After all, the median number of speakers of a language is approximately seven thousand, all of whom tend to live in relatively confined locales [5]. The WALS locations were selected to represent well the geographic centers of such locales [9].

The languages are categorized into two groups for the purpose of the present study. The first group is comprised of those languages with ejective phonemic consonants (n = 92), and the other consists of all remaining languages (n = 475) in the data set. Our grouping of languages into these two categories is derived from Maddieson’s more detailed categorization, which includes the pertinent information on basic ejective status. Just over 16% of the languages in the sample contain ejectives.

At the coarsest level, Figure 1 reveals that there is a discernible visible correlation between the six aforementioned major regions of high altitude and the locations of languages with ejective consonants. We see as well that there are eight visual clusters of languages with ejectives, highlighted via white rectangles. Two of the largest of these are located within the North American cordillera. Another is located immediately to the east of the cordillera, on the associated Colorado plateau. A fourth cluster is located just southeast of Mexican altiplano. A fifth cluster is located on the southern African plateau. The sixth and seventh clusters are located along the East African rift, on two areas of the plateau associated with this rift. The eighth cluster is located in the region of the Caucasus mountains and the Javakheti Plateau. In addition, a glance at South America reveals that a number of the languages with ejectives on that landmass are located in the Andean cordillera or on the Andean altiplano in Bolivia, as Maddieson has noted [7]. In Figure 1 a dashed rectangle highlights two proximate languages with ejectives spoken on the altiplano, to underscore this Andean bias. Remarkably, then, the clusters of languages with ejectives tend to be located on or very near five of the six major non-contiguous regions of high elevation on the earth’s inhabitable surface. The only major region of high elevation where languages with ejectives are absent is the large Tibetan plateau, along with adjacent regions of high altitude. It is not particularly surprising that one region should present such an exception, and in fact it strikes us as remarkable that only one region presents an exception.

So we can state that visible clusters of languages with ejectives are without exception located at or near one of the major regions of high elevation. Conversely, some of the richest areas of the world linguistically, in terms of languages and linguistic stocks, are largely devoid of languages with ejectives. The areas in question are Oceania (including New Guinea and Australia), Southeast Asia, West Africa, and Amazonia. Notably, all of these dense linguistic areas lack major regions of high elevation. In short, a visual analysis of the worldwide distribution of ejective languages suggests they are located at or near prominent areas of high elevation, and are markedly absent in large regions of low elevation, even though many of the latter regions are linguistically dense.

Such a coarse approximation is suggestive but inconclusive. To analyze the data in a more fine-grained manner, we ascertained the distances of all of the language locations from the nearest boundary of a major landmass exceeding 1500 m in elevation (regardless of the landmass in question). These values were derived via the distance and elevation measurement tools in Google Earth. A standardized approach to measurement was adopted, whereby an elevation map was consulted to find the closest regions of high elevation. The distance between a given language and these regions was then tested, and the shortest obtained distance was tabulated in the case of each language location. Approximately half the distance figures were tabulated by someone besides the author, a second data collector trained with this method. This second distance examiner was unfamiliar with the hypothesis being tested. No significant discrepancies were found in a contrast of the distances obtained by the author and the second data collector, when they analyzed the same set of sample data points. In short, the methods were found to be reliable across data collectors, yielding consistent distance measurements.

Remarkably, 57 of 92 (62%) languages with ejectives are located in high elevation ‘zones’, which are defined here as major regions greater than 1500 m in altitude, plus land within 200 km of such a region of high altitude. This finding is in itself surprising since, once again, only about 15% of the world’s inhabited surface area can be described as being at high elevation. In contrast, only 96 of 475 languages (20%) without ejectives are located in high altitude zones, i.e. in a major region greater than 1500 m in elevation or within 200 km of such a region. If, for the moment, we treat language locations as independent data points, we find that the disparity in the distribution of the two language groups is significant. This is apparent in Table 1.

**Table 1:** Distribution of languages with respect to regions of high elevation.

	≤200 km from 1500+ m	≥201 km from 1500+ m
Languages with ejectives	57	35
Languages without ejectives	96	379
*p*<0.0001, Two-tailed Fisher’s exact test		

Even more remarkably, 80 of 92 (87%) languages with ejectives are located within 500 km of a region exceeding 1500 m. In contrast, only 202 of the remaining 475 languages (43%) are so located. As we see in Table 2, this disparity too is highly significant. Another way to frame these results is to note that only 12 of 285 (4%) languages located further than 500 km from high elevation contain phonemic ejectives.

**Table 2:** Another breakdown of the distribution of languages with respect to regions of high elevation.

	≤500 km from 1500+ m	≥501 km from 1500+ m
Languages with ejectives	80	12
Languages without ejectives	202	273
*p*<0.0001, Two-tailed Fisher’s exact test		

Clearly languages with ejectives evince a marked tendency to occur at or near areas of high elevation. One could object, however, that this tendency in the overall distribution may be due to the location of particular linguistic families or areas that happen to have ejectives. Such familial bias could lead to autocorrelation between data points (Galton’s problem). For instance, the fact that many languages of the Pacific Northwest have ejectives and are also located at or near high elevation could yield an overall impression of geographic influence that is merely epiphenomenal. In a similar vein, ejectives could just happen to be characteristic of certain language families that are coincidentally located in high elevation zones. Such objections would be difficult to maintain, however, if numerous language families were represented by the languages with ejectives in high elevation zones, and if such languages were clustered in many diverse geographic regions. To adopt the most conservative perspective towards the data, then, we carefully considered the locations of the eight clusters of languages with ejectives, highlighted in Figure 1, and found the mean geographic center of each of these clusters (i.e. the mean latitude and longitude of the languages in a given cluster). Crucially, the mean center of seven of the eight clusters of languages with ejectives occur within high elevation zones. This distribution is also significant, as evident in Table 3. In the table, languages without ejectives are treated as clusters also, by assuming clusters contain ten languages each, in keeping with the approximate size of the clusters of languages with ejectives. In this way we treat the results as conservatively as possible vis-à-vis our hypothesis, by assuming that the overwhelming pattern in Table 1 is the by-product of the distribution of a much more modest number of regional clusters of languages that happen to share phonetic characteristics due to areal influence. Even if this simplifying assumption is made, however, the distribution of clusters of languages with ejectives is striking. We should note as well that the lone exception, in which the mean geographic center of a cluster of languages with ejectives occurs further than 200 km from an area higher than 1500 m, is for a cluster on the southern African plateau that is only marginally further away from high elevation, at 380 km. Tellingly, the geographic center in question is itself located at a relatively high elevation of 1100 m.

**Table 3:** Distribution of language clusters with respect to regions of high elevation.

	≤200 km from 1500+ m	≥201 km from 1500+ m
Clusters with ejectives	7	1
Clusters without ejectives	10	38
*p* = 0.0006, Two-tailed Fisher’s exact test		

The distribution in Table 3 is particularly remarkable given that the eight clusters in question are all geographically non-contiguous. They are separated by thousands of kilometers and oceans in many cases. Yet they all occur at high elevation or immediately adjacent to high elevation zones. Clearly, then, the marked tendency for languages with ejectives to occur in high elevation zones is not merely due to the distribution of such languages in one or a few language areas. It is also not simply the result of the undue influence of any particular language family or subset of language families, since all of the clusters of languages with ejectives represent multiple language families. More generally, the languages with ejectives in high altitude zones represent myriad language stocks including Southern Khoisan, Central Khoisan, Caucasian, Athapaskan (Na-Dene), Semitic (Afro-Asiatic), Lezgic (Nakh-Daghestanian), Armenian, Aymaran, Hadza, Mayan, Salishan, Cahuapanan, Quechuan, Siouan, Cushitic (Afro-Asiatic), Nilo-Sharan, Oto-Manguean, and Eyak (Na-Dene).

On the North American landmass (including Central America), 27 of 38 (71%) languages with ejectives occur in high elevation zones. For languages without ejectives in that same landmass, the ratio is smaller at 26 of 47 (55%). The disparity is even more apparent on other continents. In the case of South America, 7 of 13 (54%) languages with ejectives are found in high elevation zones, in contrast to 21 of 63 (33%) languages without ejectives. In Africa, 12 of 21 (57%) languages with ejectives occur in high elevation zones, whereas only 5 of 106 (5%) languages without ejectives do. Finally, in Eurasia, 11 of 13 (85%) languages with ejectives occur in a high elevation zone, while only 44 of 133 (33%) of the remaining languages do. It is worth re-stressing as well that languages with ejectives falling outside high elevation zones tend to occur very close to such zones, since worldwide only 12 of 92 (13%) languages with ejectives are located further than 500 km from regions of high elevation. In contrast, 273 of 475 (57%) languages without ejectives are located further than 500 km from major regions exceeding 1500 m in elevation.

**Figure 2:**
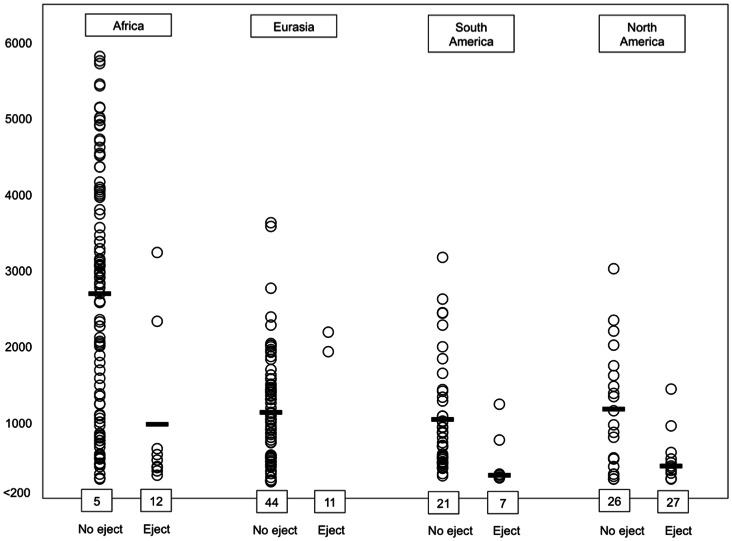
Distances (in km) of language locations from regions of high elevation. Dark lines represent means for only those languages outside high elevation zones. Numbers at the bottom of each column represent total languages within high elevation zones, i.e. within 200 km of a region higher than 1500 m. Findings for individual languages are available from the author.

Clearly, there is a marked cross-group disparity in terms of how proximate languages are vis-à-vis major inhabitable regions at high elevation. To more clearly appreciate this disparity, we examined in greater detail the locations of the languages on the four major landmasses. In the case of such languages with ejectives outside high elevation zones, the mean distance to a high elevation region is 788 km (n = 28). In contrast, the mean distance for languages without ejectives outside high elevation zones is 1937 km (n = 253). This disparity is highly significant. (t = 3.63, df 279, *p*<.0001). As we see in Figure 2, this inter-group difference is not simply due to the distribution of languages on any one particular landmass. In the case of each landmass, the languages with ejectives outside high elevation zones represent multiple language families and disparate geographic areas. Despite this heterogeneity, languages with ejectives outside high elevation zones were generally closer to such zones when contrasted to languages without ejectives. We should note that, in adopting a conservative approach to the data, we considered the distance of language locations from any clear major inhabited region of high elevation, not just the six principle regions of high altitude outlined in above. For instance, many of the distances for languages without ejectives in Europe were calculated with respect to the Alps or the Anatolian plateau. It is worth mentioning that, with respect to Africa, we did not consider the Atlas mountains to be a major region of high elevation, since the inhabitable area above 1500 m is comparatively minor when contrasted to the other African regions of high elevation mentioned above, and since the range is separated from the bulk of African languages by a major geographic barrier (the Sahara). This decision had little impact on the overall African analysis, since the disparate geographic distribution of languages with ejectives and without is so overwhelming on that continent.

The typical distance from a high elevation region was much greater for languages without ejectives, regardless of continent, even when only those languages outside high elevation zones were considered. Note that in the case of the Eurasian landmass, all languages with ejectives are located in the region of the Caucasus mountains. There are only two exceptions to this pattern, which are quite distant from high altitude zones. Interestingly one of these exceptions is a language, Korean, in which the status of ejectives is actually dubious [7].

We do not consider data from other regions in this portion of our analysis since languages with ejectives are not typically found anywhere distant from the zones of high elevation on the four principal landmasses. Since the association in question surfaces for all four of these landmasses, the tendency for languages with ejectives to occur near high elevation zones is obviously not a simple by-product of the distribution of languages on any one continent. Table 4 presents the results of t-tests contrasting the distances from regions exceeding 1500 m in elevation, for all those languages outside high elevation zones. Eurasia is not included in the table since only two languages with ejectives on that continent occur outside high elevation zones. The results for South America approach statistical significance, despite the fact that only six languages with ejectives are located outside high elevation zones on that continent.

**Table 4:** Mean distances from major regions >1500 m in elevation, for languages outside high elevation zones (i.e. ≥201 km from regions at 1500+ m).

	Africa	S. America	N. America
With ejectives	975 km	304 km	428 km
Without ejectives	2694 km	1035 km	1174 km
	t* = *3.11	t* = *1.69	t* = *2.46
	df 108	df 46	df 30
	*p* = .002	*p* = .099	*p* = .019

While we are interested in a worldwide association between ejectives and high altitude, the consideration of data from one landmass can be elucidative. We feel this is particularly true in the case of Africa, since there are several clusters of languages with ejectives on that continent, and since there are four principal areas of high elevation located on the continent’s two major plateaus. In contrast, consider that there is one principal region of high altitude in each of North America and South America, respectively, stretching primarily along a north-south axis, with languages deviating from this axis principally in terms of longitude. In the case of Eurasia, the regions of high altitude stretch primarily along an east-west axis. In a sense, then, Africa is the clearest test case for the claim that languages with ejectives tend to be located near regions of high altitude, since there are more ways in which such languages can deviate spatially from such regions given the size and placement of the high altitude zones on the continent. Despite this fact, however, it is clear from Figure 2 and Table 4 that languages with ejectives in Africa generally occur near regions of high elevation. In fact, the distribution of languages evident in Figure 2 suggests that the association between languages with ejectives and high elevation is most pronounced on the African continent. There are only two African languages in the sample (Hausa and Kotoko) that are located more than 1000 km from a region of high elevation. These two central African cases are visibly discernible in Figure 1. In that figure, it is readily apparent how they are isolated vis-à-vis the bulk of African languages with ejectives, which are generally clustered near high elevation regions. More specifically, they are clustered near high-elevation regions (3) and (4) in our list of major high elevation regions offered above. (The regions are evident in the plot of high elevation polygons offered in the inset of Figure 1.).

The clear correspondence between the locations of African languages with ejectives and major regions of elevation greater than 1500 m is particularly striking given that only a modest portion of Africa’s landmass is at such high altitude, and given that the regions of high elevation are comparatively scattered when contrasted to the major regions of high elevation on the other three major landmasses. Furthermore, each of the three African clusters of languages with ejectives represents multiple language families. In short, Africa offers a compelling illustration of the strong worldwide association between areas of high elevation and the usage of phonemic ejectives.

To this point, we have focused on the location of languages with respect to regions of high elevation. We also ascertained the actual elevation of each language point in the data set, including those outside the four major landmasses. We should note that these elevation figures are generally conservative with respect to our hypothesis, since many language locations are near regions of high elevation but are given low elevation scores. Most notably, in the Pacific Northwest many languages with ejectives were found to be at low elevation since their coordinates occur near the ocean. So not surprisingly the elevation figures for languages in this region, in which ejectives are a common feature, were often found to be low–despite the fact that the speakers of these languages also subsist in nearby mountainous areas, not just along the coast. Despite this conservative influence on our data, however, a significant difference was found between the elevations of languages with ejectives and those without. The mean elevation for all languages without ejectives was 631 m. (This does not imply that most people live at this elevation, since many of the world’s most-widely spoken languages like English and Spanish have low elevation values but are only considered singular data points in such an analysis.) In the case of languages with ejectives, the mean elevation was 955 m, a full 51% higher. This difference was significant. (t = 3.84, df = 565, *p = *.0001) In the case of languages without ejectives, the median elevation was 340 m. (This figure is nearly half that of the mean elevation for this group, in part since the mean elevation is influenced by outliers in the Himalayas.) In the case of the languages with ejectives, the median elevation was 668 m, a full 96% greater than the median of the remaining languages. While elevations differed on a by-continent basis, the disparity exhibited by the two language groups was clearly not simply the result of their distribution in any one major region, as we see in Table 5.

**Table 5:** Mean elevations of language locations.

	World	Africa	Eurasia	S. America	N. America
With ejectives	955 m	1203 m	1449 m	967 m	750 m
Withoutejectives	631 m	634 m	800 m	452 m	769 m
	t* = *3.84	t* = *4.10	t* = *2.12	t* = *2.09	t* = *.119
	df 565	df 125	df 144	df 74	df 88
	*p*<.00001	*p*<.0001	*p* = .035	*p* = .041	*p* = .91

**Figure 3:**
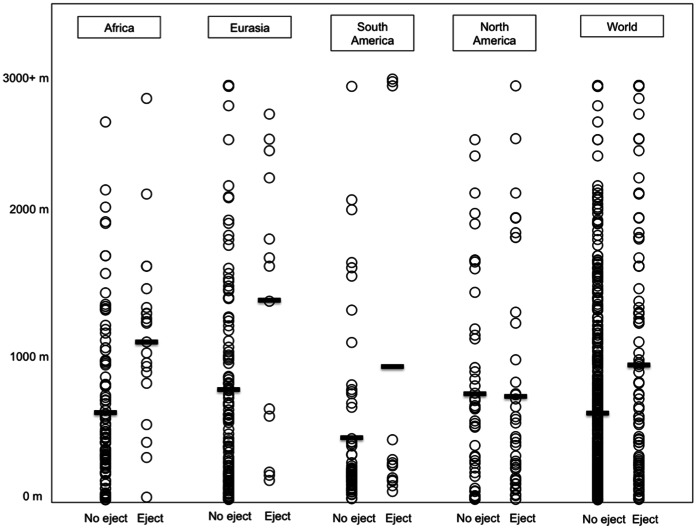
Plot of the elevations of language locations, for all 567 languages in the sample. Means are highlighted with dark lines. Findings for individual languages are available from the author.

The only case in which there is not a significant disparity in the elevations of the two language groups is the North American landmass. As we observed in Figure 2 and Table 4, however, languages with ejectives on that landmass do occur closer to high elevation zones at a significantly greater rate, when contrasted to languages without ejectives. Given that the elevation of so many ejective languages in the Pacific Northwest is taken from points near the sea level, however, it is not surprising that no noticeable disparity is observed between the two language groups on this landmass, in terms of absolute elevation. In addition, it is worth noting that there are a number of languages without ejectives at high elevation on the Mexican altiplano. Again, though, in terms of location vis-à-vis high elevation zones, we have already found a robust difference between the languages with and without ejectives on the North American landmass. This difference in locations was observed for all four major landmasses, and the elevation figures offered in Table 5 further corroborate the worldwide correlation uncovered. This worldwide correlation is also apparent in Figure 3, in which we have plotted the elevations of all the language locations in our database, according to the landmass on which they occur. Note that the plots of the data points in the column labeled ‘World’ represent all 567 languages, including those from Australia, New Guinea, Indonesia, Melanesia, Polynesia, and elsewhere.

**Figure 4:**
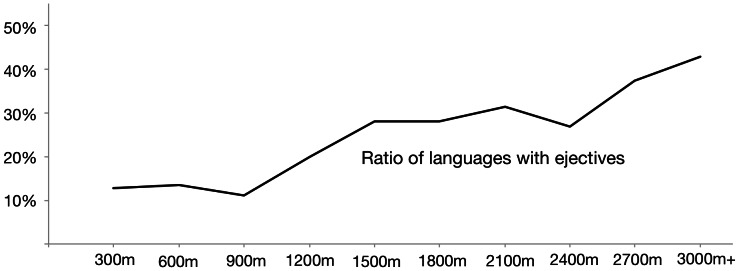
Percentage of languages with ejectives, categorized according to the elevation at which the languages are spoken.

Perhaps the most remarkable facet of the elevation data gleaned from our analysis is presented in Figure 4. As we see in the figure, as elevation increases so does the likelihood that a language found at that elevation utilizes phonemic ejectives. Figure 4 is also based on all of the 567 languages in the data sample. (See Data Set S1 for the elevation of each language with ejectives.).

Given the robust nature of the relationship between the locations of languages with ejectives and high elevation zones, which is observed on a global scale, we are left to conclude that there is some underlying motivation for this correlation, which clearly cannot be explained in terms of the influence of particular linguistic families or in terms of the coincidental spread of ejectives across languages in particular regions. In the following section we offer two plausible motivations for the correlation, and then discuss the implications of our finding.

## Discussion

The vast majority of the sounds of the world’s languages are made via pulmonic airflow passing through various configurations of the human vocal tract. While three sound categories are non-pulmonic–namely clicks, implosives, and ejectives–only ejectives entail the use of egressive non-pulmonic air. Both implosives and clicks are made by creating rarefaction in the mouth, which results in ingression of extra-oral air that is at comparably higher pressure. Implosives, like ejectives, involve the movement of the glottis and are also considered ‘glottalized’ sounds, all of which are generally considered articulatorily complex [7]. Unlike ejectives, the glottis moves downward in implosives, and the vocal cords are not generally closed completely [6]. During the production of ejective sounds the glottis is closed and, typically, raised. The unique articulatory characteristic of ejectives is that they involve the compression of air in the pharyngeal cavity, air which is ejected after the release of occlusion at some place in the vocal tract forward of the pharyngeal cavity. The most common place of articulation of this forward location is the velum. Conclusions vary as to why velar ejectives are so common, with a general consensus being that ejectives are highly distinctive perceptually at the velar place of articulation, when contrasted with simple pulmonic plosive consonants made at the velum [6], [10]. Other factors include the possibility that it is easier to constrict the pharyngeal cavity by a general upward motion of the glottis and simultaneous tongue-to-velum movement [11]. The amount the glottis is raised during the production of ejectives appears to vary across languages [12], [13], and there are other minor articulatory variances between ejectives across the languages that have them [12]. Nevertheless, what is common to all phonemic ejective sounds is that the pharyngeal cavity (and a portion of the oral cavity in some cases) is constricted, and air from the pharyngeal cavity (and potentially oral cavity) is compressed and released through the mouth.

Since ejectives are made via the compression of air supra-glottally, we initially speculated that their articulation might be facilitated at higher elevations, since atmospheric air pressure (and therefore the air pressure inside one’s mouth and lungs) is reduced at such elevations. The grounding for our hypothesis follows naturally from some basic principles of air pressurization.

The pressure differential created by a velar ejective sound, the most common kind of ejective, can be schematically described by subtracting the air pressure in the pharyngeal cavity prior to constriction (P1) from the pressure after constriction (P2), i.e. P2-P1. P2 can be found via Boyle’s law: P2 = (V1×P1)/V2. In other words, the air pressure differential (P2-P1) is created by compressing the pharyngeal air cavity (V1) at a given atmospheric pressure (P1), by reducing the cavity to a smaller volume (V2). For instance, we may reasonably assume that the volume of a human pharyngeal cavity is 40 cm^3^, in accordance with typical estimates. During the production of a velar ejective sound, that volume might be reduced by 2 cm^3^ (or more) to 38 cm^3^. If the ejective is being produced at sea level, at which atmospheric air pressure is typically about 1030 cm H2O, P2 will be 1084 cm H2O. [(40 cm^3^×1030 cm H2O)/38 cm^3^]. The pressure differential created by the ejective would be 54 cm H2O (1084 cm H2O-1030 cm H2O). Now let us assume that a velar ejective is made at a higher elevation, for instance 2500 m. At such an elevation, the atmospheric pressure is typically around 760 cm H2O. The pressure differential created by the same articulatory motion in this case will be less, assuming that the pharyngeal cavity is once again compressed to 38 cm^3^. In fact, the pressure differential in such a case would be 40 cm H2O, the difference between P2 (800 cm H2O) and P1 (760 cmH2O). In other words, the relevant ejective pressure differential at 2500 m would be 14 cm H2O less than the pressure differential produced at sea level with the identical articulatory gesture. (54 cm H2O-40 cm H2O) Since force is simply a matter of the pressure exerted on a given area, it follows that the force required to produce the articulatory gesture at 2500 m would be roughly 26% (14 cm H2O/54 cm H2O) less than the force required at sea level. In short, this basic modeling predicts that the articulatory act of compressing the volume of air in the pharyngeal cavity should be easier at higher altitude, since less compression force must be produced via the exertion of the stylohyoid and digastric muscles contracted during the raising of the glottis. This articulatory facilitation might help to motivate the preponderance of ejectives at altitude. Facility of articulation is already known to be a factor in the commonality of many sounds across the world’s languages, for instance bilabial and alveolar plosives.

**Figure 5:**
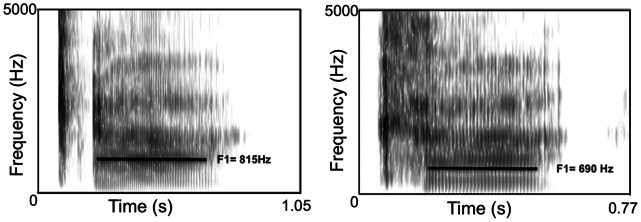
Spectrograms of [k’a] (on the left) and [k^h^a]. Note the relatively high first formant of the vowel following the velar ejective consonant in the first spectrogram.

While this account is not implausible, it is open to at least one objection. One of the clearest acoustic correlates of ejective sounds is a characteristic aperiodic burst of egressive air [14], and it seems that the salience of this burst of air would be reduced in cases of lower atmospheric air pressure. In other words, the aforementioned lower pressure differential would in theory make ejectives easier to produce but also less perceptually salient at higher altitudes. While this objection is not without merit, though, it may oversimplify matters. After all, like all plosive consonants, the perceptual salience of ejectives is largely a result of their impact on adjacent vowel formants [15]. Given that ejectives involve the constriction of the pharyngeal cavity, the first formant of adjacent vowels is typically increased by the ejective articulation. (The first formant is essentially the pharyngeal harmonic resonance of the frequency of vibrating vocal cords.) For instance, in our own recordings of the syllable [k’a], the first formant of the vowel following the velar ejective consonant is typically 100–150 Hz higher than the first formant of the vowel following the aspirated velar plosive in [k^h^a]. This disparity is evident in the two spectrograms depicted in Figure 5, based on recordings made via PRAAT acoustic analysis software [16] with a sampling rate of 44.1 kHz.

Given that one of the key acoustic characteristics of ejectives is their impact on the acoustic structure of adjacent vowels, it seems quite possible that they are preponderant at high altitudes due in part to articulatory ease, even though lower atmospheric pressure might reduce the salience of their associated burst of air. We recognize that this issue is not resolved, but believe this account sheds light on how low atmospheric pressure might directly impact the ease of articulation of ejectives, resulting in the tendency for these sounds to be more common in areas of high altitude.

We would also like to offer a second explanation of the preponderance of ejectives in high elevation zones. The second account relates to human biology in a more basic manner. Like aspirated plosives, ejective sounds have an aspiration-like burst of air, but one that does not require a release of air from the lungs to achieve. While air from the lungs must obviously be released at any elevation for the sake of respiration, the manner in which it is released during speech varies in accordance with the rate at which the glottis is closed while a person speaks, and in accordance with the ratio of egressive pulmonic sounds used. If the glottis is closed for a substantial portion of the sounds in a given language and if egressive pulmonic sounds are relied upon less extensively (as in a language that relies on a class of ejective consonants), less pulmonic air should be required for actual speech production. As a result, exhaled minute ventilation, the amount of air exhaled from the lungs per minute, could be reduced somewhat during speech that relies heavily on ejective sounds. Significantly, minute ventilation correlates positively with the amount of exhaled breath condensate (EBC) released during breathing [17]. EBC is composed of “droplets of airway surface liquid diluted by water vapor” [17]. In other words, there is a positive correlation between minute ventilation, which during speech should theoretically be mitigated directly by the usage of ejectives, and the amount of EBC and water vapor lost.

This latter point is hardly trivial. Humans typically lose 300–400 ml of water vapor daily through exhaled breath, since expired air contains approximately 6% water vapor. This amount is increased at higher elevations with characteristically low rates of ambient water vapor in the air. The source of water vapor in expired air is the respiratory mucosa, for instance the tracheal walls that have been demonstrated to release water moisture [17].

Dehydration is achieved more readily at higher elevation due to the aforementioned low ambient water vapor, and is a major factor in motivating altitude sickness (in the colloquial sense of the term, not in the strict sense of hypobaropathy). Preventative hydration is often recommended when people visit altitudes over 1500 m. In the light of these factors, we would like to suggest the possibility that ejectives may reduce water vapor lost through exhalation. This suggestion is likely to meet with some skepticism, but we see no reason to immediately doubt it. We are simply suggesting that heavy reliance on ejective consonants may yield less EBC lost during speech, thereby promoting the retention of water vapor. Obviously, exhaled minute ventilation is principally the result of respiratory needs, and pulmonic air must be exhaled regardless of the language a person speaks. Nevertheless, pervasive usage of egressive pulmonic sounds would seem to promote higher rates of exhaled minute ventilation. One tip given to mountain climbers seeking to avoid water vapor loss is, after all, to simply speak less.

This second proposal, namely that ejectives mitigate water vapor loss, would appear to be buttressed by the fact that human populations at high elevations are already adapted biologically and behaviorally in numerous ways to their dryer ambiences with less oxygen density. For instance Tibetan and Andean populations exhibit distinct genetic adaptations that reduce the effects of hypoxia, and Tibetan populations breathe at a faster rate than tested control populations [18], [19]. (It is interesting to note that the one major region of high elevation without languages with ejectives is the Tibetan plateau, an area in which people have adapted to high altitude in distinct ways vis-à-vis respiration [19].) So on some level it seems intuitive that such adaptations could surface in the linguistic realm. Nevertheless, we admit that this account is far from dispositive. We are merely describing one biologically motivated functional mechanism through which ejective consonants might be favored at high altitudes, as they potentially reduce the amount of water vapor lost during speech. We recognize that a battery of experiments would need to be carried out to test this hypothesis, but the hypothesis itself is reasonable in the light of the physiology of the human respiratory and vocal tract.

Finally, it is possible that both of the factors we have suggested may operate in concert, so that ejectives are favored at high elevations because they are easier to articulate in such locales, *and* because they attenuate (however moderately) the rates of water vapor loss in exhaled breath. It is also possible that neither account is correct. Nevertheless, what is now clear is that there is a worldwide correlation between a geographic factor, elevation above sea level, and a phonological factor, likelihood of a language relying on ejective phonemes. In the light of this association, we have offered two reasonable yet tentative explications of the correlation, both of which illustrate how elevation might have come to impinge upon the sounds in human languages.

### Conclusions

The evidence we have adduced clearly supports the following conclusion: Languages spoken at higher altitudes are significantly more likely to rely on ejective phonemes. This conclusion is supported by data from every major world region. Languages with ejectives are generally absent in vast linguistically dense regions at low elevations. Despite the fact that the great majority of languages do not utilize ejective phonemes, however, languages with ejectives are quite common in regions of high altitude. This distribution does not owe itself simply to the influence of language families or the homogenizing effects of particular linguistic regions. Ejectives have spread across languages in numerous areas in high elevation zones. This is not to suggest that languages with ejectives do not occur in areas far from high elevation zones. There are twelve such cases in our data sample. What is striking is that in those cases ejectives have not spread to surrounding languages. In contrast, in areas of high altitude ejectives are in numerous cases a regional feature, and these articulatorily complex sounds have spread across many languages of distinct linguistic stocks in such areas. Interestingly, on the Tibetan plateau and in adjacent regions, ejectives are altogether lacking in our data set. Given that no ejectives seem to exist in the region, ejectives have obviously not been able to spread within the area. This region would be more exceptional, given the distribution of languages with ejectives observed elsewhere, if only one or a few such languages were observed in the region.

Given the established association between an aspect of geography and linguistic form, we are left to speculate why such a relationship might exist. We have offered two initial explanations of the correlation, both of which (or neither) may be operative in motivating the predilection for phonemic ejectives at altitude. The acceptance of our major finding is not contingent on either of these explanations. Nevertheless, the plausibility of these explanations sheds light, we believe, on how the correlation we have uncovered might have come to exist.

It is well known that languages adapt to their local ecologies lexically, e.g. they innovate terms for flora and fauna that are specific to an ecological niche, or develop lexically in accordance with social and technological shifts. In addition, other associations between lexicons and environment have been observed, for instance the distinction between a word for ‘arm’ and ‘hand’ in a given language is less common in warmer climates in which people are generally less clothed [20]. In a related manner, some other language-external factors have also been claimed to impact language form–recent evidence suggests that the size of a linguistic population may impact the relevant language’s morphological complexity [21]. Other studies also suggest that population size may correlate with the size of a language’s phonemic inventory [22], [23], [24], though some linguistic typologists have explicitly rejected this hypothesis [25]. The possibility that geographic factors such as altitude might directly influence phonology has not been systematically explored until now. (Though the possibility has occasionally been mentioned in passing, e.g. in [26], it has more generally been satirized–see the relevant discussion of linguists’ blogs in [27].).

Certainly a guiding assumption for many linguists is that languages are not shaped in any structural, non-lexical ways by external factors, and it is generally assumed that they are not shaped in such ways by geographic factors such as elevation. What we are suggesting here is that, contra common presumptions, language structure is in fact impacted by geography, at least in the manner described here. We have presented evidence for a direct sort of geographic influence on arguably the most basic aspect of the form a given language, its inventory of sounds.

## Supporting Information

Data Set S1 **Coordinates and elevations of locations of languages with ejectives.**
(PDF)
