# Supplementary material for: Evidence for Direct Geographic Influences on Linguistic Sounds: The Case of Ejectives
Source: PLoS One. 2013 Jun 12;8(6):e65275. doi: 10.1371/journal.pone.0065275 (PMC3680446; doi:10.1371/journal.pone.0065275)
Supplement: Data Set S1 — Coordinates and elevations of locations of languages with ejectives. (PDF) [file pone.0065275.s001.pdf]

Coordinates and elevations of locations of languages with ejectives

Language	Longitude	Latitude	Elevation
//Ani	21.917	-18.917	993
Abkhaz	41	43.083	202
Ahtna	-145	62	731
Amharic	38	10	1532
Archi	46.833	42	2794
Armenian	45	40	1890
Avar	46.5	42.5	1755
Aymara	-69	-17	3970
Tsova-Tush	45.5	42.5	2619
Bella Coola	-126.667	52.5	267
Berta	34.667	10.333	1367
Caddo	-93.5	33.333	82
Chehalis	-123	46.583	116
Coos	-124.167	43.5	40
Dizi	36.5	6.167	845
Eyak	-145	60.5	10
Georgian	44	42	607
Hadza	35.167	-3.75	1292
Hamtai	146.25	-7.5	984
Huastec	-99.333	22.083	586
Hunzib	46.25	42.167	2529
Hupa	-123.667	41.083	416
Ingush	45.083	43.167	661
Itelmen	157.5	57	140
Itonama	-64.333	-12.833	137
Jakaltek	-91.667	15.667	2041
Jaqaru	-76	-13	3064
Jebero	-76.5	-5.417	269
Ju|'hoan	21	-19	1066

Language	Longitude	Latitude	Elevation
Kefa	36.25	7.25	1695
Kiowa	-99	37	558
Korean	128	37.5	183
Lak	47.167	42.167	1442
Lakhota	-101.833	43.833	768
Lezgian	47.833	41.667	1694
Makah	-124.667	48.333	10
Navajo	-108	36.167	1903
Chulupí	-60.5	-23.5	143
Pomo	-122.5	39	459
Qawasqar	-75	-49	409
Quechua	-66	-17.5	3435
Quileute	-124.25	47.917	237
Rutul	47.417	41.5	2339
Sandawe	35	-5	1280
Selknam	-70	-53	66
Shasta	-122.667	41.833	596
Siona	-76.25	0.333	256
Tehuelche	-68	-48	102
Tigré	38.5	16.5	1354
Tiwa	-105.5	36.5	3291
Tlingit	-135	59	443
Tol	-87	14.667	1281
Trumai	-53.583	-11.917	292
Tzeltal	-92.5	16.417	761
Wintu	-122.5	41	853
Yeyi	23.5	-20	952
Zuni	-108.833	35.083	1934
!Xóõ	21.5	-24	1144
Acoma	-107.583	34.917	1903
Haida	-132	53	161

Language	Longitude	Latitude	Elevation
Klamath	-121.5	42.5	1362
Kutenai	-116	49.5	2225
Kwakw'ala	-127	51	85
Wichí	-62.583	-22.5	250
Nez Perce	-116	46	674
Nuuchahnulth	-126.667	49.667	394
Shuswap	-120	52	1014
Slave	-125	67	320
Soqotri	54	12.5	707
Squamish	-123.167	49.667	74
Tsimshian	-129	52.5	10
Wappo	-122.5	38.5	225
Wichita	-97.333	33.333	253
Yapese	138.167	9.583	22
Yuchi	-86.75	35.75	280
Yurok	-124	41.333	118
Lushootseed	-122	48	149
Mazahua	-99.2	19.41	2619
Nambiquara	-59	-13	436
Dahalo	40.5	-2.33	21
Deti	24.5	-20.5	925
Hamer	36.5	5	966
Hausa	7	12	546
Iraqw	35.5	-4	2217
Zulu	30	-30	1313
Kekchi	-89.83	16	141
Komo	33.75	8.75	0
Kotoko	15.33	11.33	417
Kullo	37.08	6.75	1694
Maidu	-120.67	40	2045
Oromo	42	9	1396

Language	Longitude	Latitude	Elevation
Yucatec	-89	20	37
